# Air-Stable Thin Films of Tin Halide Perovskite Nanocrystals by Polymers and Al_2_O_3_ Encapsulation

**DOI:** 10.1021/acs.chemmater.4c02261

**Published:** 2024-11-15

**Authors:** Kushagra Gahlot, Lorenzo di Mario, Rixt Bosma, Maria A. Loi, Loredana Protesescu

**Affiliations:** Zernike Institute for Advanced Materials, University of Groningen, Nijenborgh 3, 9747 AG Groningen, The Netherlands

## Abstract

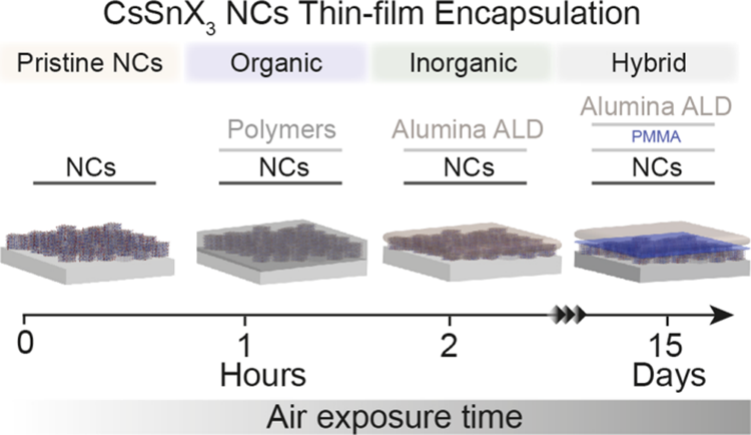

Tin halide perovskites are promising for optoelectronics, although their sensitivity to ambient conditions due to Sn(II) oxidation presents a challenge. Encapsulation techniques can mitigate degradation and facilitate advanced studies of the intrinsic properties. To study and improve the ambient stability of CsSnBr_3_ and CsSnI_3_ nanocrystal (NC) thin films, we explored various encapsulation methods: organic, inorganic, and hybrid. We employed three methods for organic encapsulation: co-deposition with NCs, co-deposition with an additional top layer, and *in situ* polymerization with NCs. We synthesized thin layers of alumina by using atomic layer deposition for inorganic encapsulation. While individual methods offered marginal improvements, the hybrid approach provided the best results. By employing a hybrid heterostructured thin-film strategy, with the NC layer covered by a thin layer of poly(methyl methacrylate) followed by a 40 nm alumina layer, the stability in air was improved from a few seconds to a record period of 15 days, a crucial advancement for the further exploration of tin halide perovskites.

## Introduction

Tin halide perovskites are emerging as a preferable lead-free perovskite material for various optoelectronic applications, especially in photovoltaic devices with up to 15% power conversion efficiencies.^1−5^ However, one of the major drawbacks of tin halide perovskites is their susceptibility to ambient environments, which stems from the oxidation of Sn^2+^ to Sn^4+^ in the presence of oxygen or moisture. Various chemical approaches such as additive engineering,^6−8^ dimensional reduction,^3,5^ and solvent engineering^9,10^ have been deployed for bulk thin-film fabrication to overcome this issue. All of these chemical approaches can further benefit from the advancement of encapsulation strategies to improve the long-term performance and stability of perovskite-based optoelectronic devices.^11−13^ For thin films obtained from tin halide perovskite nanocrystals (NCs), the issue of instability escalates or worsens due to a larger surface area exposed to the ambient environment and the difficulty in maintaining the morphology with confinement properties.^14,15^ Thus, a thoughtful encapsulation strategy is required to enhance the air stability of tin halide perovskite NC thin films.

When tin halide perovskites are exposed to air, the desired crystal phase of CsSnX_3_ quickly oxidizes, causing self-doping (oxidation to Sn^4+^), which deteriorates their optoelectronic properties. For instance, the γ-orthorhombic phase of CsSnI_3_ which exhibits excellent photovoltaic properties degrades to zero-dimensional Cs_2_SnI_6_.^16,17^ Organic encapsulation of bulk perovskite thin films with polymers is one of the most effective large-scale, low-cost alternatives to achieve long-term stable devices shielded from the ambient environment.^13,18^ On the other hand, for inorganic encapsulation of bulk and NC thin films with metal oxides such as alumina (Al_2_O_3_),^19−22^ silica (SiO_2_),^23,24^ nitrides like silicon nitride (Si_3_N_4_),^25^ and metal halide salts,^26^ to name a few, high-vacuum deposition techniques or simple solution methods were used. Kiyek *et al.* deposited CsSnI_3_ with a capping layer of Al_2_O_3_ via pulsed layer deposition, showcasing that the thin films are stable in air for 72 h.^27^ Hybrid organic and inorganic encapsulation has been successfully employed in technologies like organic solar cells^6,28,29^ and organic light-emitting diodes.^30,31^ Along the same direction, for improving the stability for both lead and tin-based perovskite solar cells, various polymers have been coupled with atomic layer deposition (ALD) Al_2_O_3_ and silicate (SiO_2_) to form a multilayer thin film that could provide higher environmental resistance.^13,29,32,33^

For thin films deposited from colloidal tin halide perovskite NCs, various polymers such as poly(styrene) (PS),^34,35^ poly(methyl methacrylate) (PMMA),^36^ poly(vinylidene fluoride) (PVDF), poly(dimethylsiloxane) (PDMS),^37^ and cyclic olefin copolymers (COCs)^38^ could be utilized to improve the chemical and optical stability of NCs based on previous reports on the analogue Pb halide perovskite NC studies. However, Gao *et al.* reported the only example of organic encapsulation of tin halide perovskite NCs (CsSnCl_3_) via bone gelatin to enhance their stability in aqueous solutions.^39^ Moreover, the limited successful reports of the inorganic and hybrid organic–inorganic encapsulation method being utilized to enhance the air stability of tin halide perovskite NCs are also due to the scarcity of synthetic studies compared to Pb-based perovskite NCs.

In this work, we employed our previously reported CsSnX_3_ colloidal NCs^40^ (X = I, Br) and utilized organic, inorganic, and hybrid organic–inorganic encapsulation methods to improve the stability of the thin film formed using drop-casting and spin-coating CsSnX_3_ NCs. Among the variety of insulating and conducting polymers we screened in this work, we chose PS, based on the final composite’s chemical structure and performance, for the polymer encapsulation of CsSnX_3_ NC thin films. Three different methodologies were employed for PS encapsulation: (i) PS deposition with NCs, (ii) deposition with an extra top layer of PS, and (iii) *in situ* polymerization. For inorganic encapsulation, Al_2_O_3_ deposited via the ALD technique was utilized at two different thicknesses (∼20 and ∼40 nm). Finally, we performed a hybrid organic–inorganic encapsulation of CsSnX_3_ (X = I, Br) NC thin films by depositing a thin layer of PMMA to increase the adhesion and stability, followed by a 40 nm Al_2_O_3_ layer via ALD. We achieved a record stability in air of over 15 days for CsSnX_3_ NC thin films with the hybrid encapsulation strategy characterized by time-dependent X-ray diffraction (XRD) and optical spectroscopy. This enhancement in the air stability of the CsSnX_3_ NC thin films is noteworthy and can pave the way for further studies and applications of tin halide perovskites.

## Materials and Methods

### Materials

Cesium carbonate (Cs_2_CO_3_, Sigma, metals basis, 99.9%), tin(II) iodide (Sigma, anhydrous beads, 99.99%), tin(II) bromide (TCI Europe), octadecene (ODE, Sigma, tech. grade, 90%), oleic acid (OA, Sigma, tech. grade, 90%), oleylamine (OLA, Sigma, tech. grade, 70%), toluene (Acros, extra dry, 99.85%), styrene (99%, extra pure, stabilized, ACROS Organics), hexamethyldisilazane (Sigma, >99%, reagent grade), azobis(isobutyronitrile) (AIBN, Sigma, 98%), poly(styrene) (PS, Styron 634, Trinseo), poly(methyl methacrylate) (PMMA, Sigma, avg mw ∼350,000), cyclic olefin copolymer (TOPAS 6013 M-07), poly(ethylene oxide) (PEO, Sigma, mw 600,000), poly(dioctylfluorene) (PFO, Ossila, F8), poly(didodecyl fluorene) (PF-12, Ossila, F12), poly(4-butyl triphenylamine) (p-TPD, Sigma, mw ≥ 20,000), and poly(9-vinylcarbazole) (PVK, Sigma, mw 25,000) were used.

### Methods

#### Synthesis of CsSnX_3_ NCs

The synthesis of three-dimensional (3D) CsSnX_3_ nanocrystals followed the procedure outlined by Gahlot *et al.*(40,41) SnI_2_/SnBr_2_ (2 mmol) along with dried oleic acid (OA) (0.63 mL, 2 mmol), oleylamine (OLA) (0.66 mL, 2 mmol), and 1-octadecene (ODE) (5 mL) was placed in a 25 mL three-neck flask within a nitrogen (N_2_) glovebox. The reaction mixture was then transferred to a Schlenk line and stirred under vacuum at room temperature for 10 min, followed by heating to 105 °C and vigorous stirring for 45 min. The temperature was then raised to 200 °C under a N_2_ flow, and 2.8 mL (0.622 mmol) of cesium oleate (0.222 M) was swiftly injected. The reaction was quenched after 10 s by rapidly immersing the flask into an ice–water bath. Subsequently, the reaction flask was carefully moved back to the glovebox for purification. The crude solution was divided equally into two centrifuge tubes and centrifuged at 5000 rpm (7082 rcf) for 3 min. The supernatant was discarded, and the precipitate was dispersed in toluene (5 mL) followed by centrifugation at 13,000 rpm (18,412 rcf) for 5 min. The supernatant was again discarded, and the precipitate was redispersed in 10 mL of toluene for further experiments.

#### Preparation of CsSnX_3_ NC/Polymer Solutions

The NC solution was filtered using a 0.45 μm PTFE filter to achieve a concentration of ≈50–55 mg/mL. Different concentrations of polymer solutions were prepared by weight/volume (1 and 2%) in toluene. The thin-film fabrication was performed by mixing the above solutions in a 1:1 ratio by volume. (For PS, 5 and 10% polymer solutions were also made since the NCs were stable at those concentrations).

#### Preparation of Glass Substrates

1 × 1 glass substrates were washed with soap water, followed by 3 cycles of ultrasonication in ethanol and then in acetone. The substrates were then completely dried with a jet of compressed air. 50 μL of hexamethyldisilazane was dropped on the clean glass substrates and annealed at 120 °C for 30 min. These substrates were then transferred to the glovebox carefully.

#### Thin-Film Fabrication

##### Organic Encapsulation

NC/PS thin films: The glass substrate was placed flat in the spin coater. 30 μL of NC/PS solution (5%) was dropped on the substrate followed by a two-step spin-coating procedure, i.e., 1000 rpm for 10 s followed by 2000 rpm for 20 s. For the topping layer of PS: After the above procedure, 20 μL of 10% PS solution was utilized with spin-coating parameters of 2000 rpm for 30 s. *In situ* polymerization of styrene: To remove the inhibitor from styrene, 8 mL of styrene was washed 3 times with 8 mL of 5% aqueous solution of NaOH. The styrene phase was then separated and 1 g of MgSO_4_ was added to absorb the remaining water. This solution was then filtered and used for polymerization with the NC solution for thin-film fabrication. The thin films were fabricated via a drop-casting method from 30 μL of CsSnI_3_ NC (≈50 mg/mL) solution prepared in purified styrene with AIBN used as an initiator, and the modified procedure reported by Tefera *et al.* was used at 80 °C.^42^

##### Inorganic Encapsulation

For alumina ALD, thin films were fabricated via a two-step spin-coating procedure mentioned above with 30 μL of 50 mg/mL CsSnX_3_ NC solution. These thin films were transferred to the ALD set up in an airtight container and loaded in the chamber for deposition.

##### Hybrid Organic–Inorganic Encapsulation

Thin films were fabricated via a two-step spin-coating procedure mentioned above with 30 μL of 50 mg/mL CsSnX_3_ NC solution. This was followed by the spin-coating of 30 μL of 2% PMMA solution/PS solution at 2000 rpm for 30 s. These thin films were transferred to the ALD set up in an airtight container and loaded in the chamber for deposition.

#### Characterization

Powder X-ray diffraction measurements were performed in an inert dome sample holder in a Bruker D8 Advance diffractometer in Bragg–Brentano geometry using Cu Kα radiation (λ = 1.54 Å) and a Lynxeye detector. The absorption measurements and steady-state photoluminescence measurements were performed on a table-top Avantes ultraviolet–visible (UV–visible) spectrophotometer using a tungsten and halogen filament lamp as the excitation source. The steady-state photoluminescence measurements were performed on a Horiba Scientific Jobin Yvon spectrometer equipped with a PMT detector. For scanning transmission electron microscope (STEM) images, the samples were prepared on an ultrathin grid with 400-mesh Cu (Ted Pella, Inc. 01822-F), which was wrapped with graphene on one side. The sample was then drop-cast on the graphene side of the grid, which was then sandwiched between two graphene layers using the other grid. The TEM grid was dried overnight in the antechamber of the glovebox. The measurements were performed on a Thermo Fisher Themis Z STEM instrument operating at 300 kV. Alumina films were deposited by ALD with a Picosun R200 advanced system. Trimethylaluminum (TMA) and H_2_O were used as precursors. The precursor bottles were kept at 20 °C during the deposition. A pulse duration of 0.1 s was used for both precursors, with purge times of 6 and 9 s after the pulse of TMA and H_2_O, respectively. The ALD chamber was kept at 60 °C during the deposition. A number of cycles of 300 and 600 were used to obtain film of alumina of 20 and 40 nm, respectively.

## Results and Discussion

### Air Degradation of the CsSnI_3_ NC Thin Film

**Figure 1:**
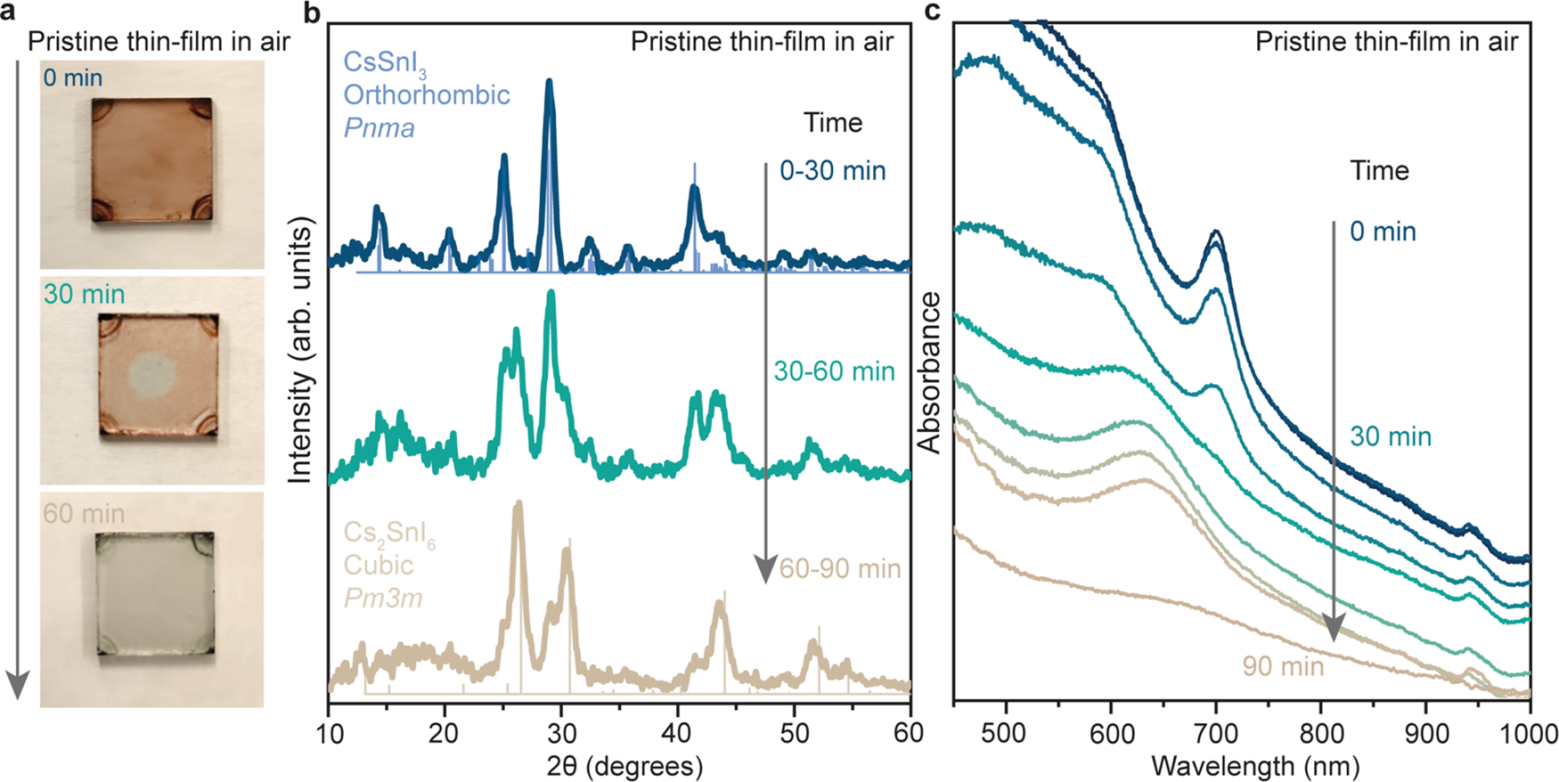
(a) Photographs of the CsSnI_3_ NC thin film, fresh and exposed in air for 30 and 60 min. (b) XRD patterns of the CsSnI_3_ NC thin films measured after exposure to air for the indicated time. (c) *In situ* UV–visible absorption spectra of the CsSnI_3_ NC thin film exposed to air for 90 min.

We synthesized tin halide perovskite NCs^40,41^ using our previously reported protocols. We deposited them on glass substrates *via* a simple two-step spin-coating method and we studied their structural and optical properties upon air exposure. Figure S1 represents the structural morphology and size of the CsSnX_3_ NCs (X, Br, I) utilized for this study. Figure 1a shows the visual degradation of the obtained CsSnI_3_ NC thin film, which changed color from dark brown to green in 60 min when exposed to air. XRD measurements performed on these thin films before and after exposure to air showed the degradation of the orthorhombic *Pnma* CsSnI_3_ crystal structure to a cubic *Fm*3*m* Cs_2_SnI_6_ crystal structure (Figure 1b). The broad peaks in the XRD pattern confirmed the presence of NCs in the thin films. Further, we measured the in situ UV–visible absorption spectra while exposing our films to ambient conditions (Figure 1c). Initially, we observed a narrow excitonic peak at 702 nm, characteristic of CsSnI_3_ NCs,^40,43^ which, upon exposure to air, decreased continuously in intensity. After 90 min, we only observed a broad signal (full width at half-maximum (fwhm) > 0.15 eV) attributed to polydispersed Cs_2_SnI_6_ nanostructures.^44−46^ The corresponding steady-state photoluminescence (PL) spectra of CsSnI_3_ NCs in suspension and thin films are shown in Figure S2. The PL peak at 720 nm quenches completely upon air exposure in 60 min for the NC solution and in 20 min for the NC thin films. Since it is clear from these experiments that encapsulation methods must be developed to suppress the oxidation of tin halide perovskite NCs, we further probed the potential of organic, inorganic, and hybrid coating techniques.

### Organic Encapsulation of the CsSnI_3_ NC Thin Film

Following strategies initially employed for lead-based halide perovskite NCs^34−38,47^ and thin films containing tin halide perovskites,^13,33,39^ we explored the encapsulation of our CsSnI_3_ NC thin film with the help of organic polymers which were either insulating (PS, PMMA, poly(ethylene oxide) (PEO), and COCs)^48,49^ or conductive (poly(dioctylfluorene) (PFO), poly-*N*,*N*′-bis(3-methylphenyl)-*N*,*N*′-diphenylbenzidine (p-TPD), poly-9,9′-di-*n*-dodecyl-fluorenyl-2,7-diyl (PF-12), and poly(vinylcarbazole) (PVK)). We initially tested the stability of CsSnI_3_ NCs in these polymer solutions (conc. ≈50 mg/mL in toluene, 1:1 by volume with polymers, Figure S3). We optimized our studies by keeping the concentration and volume of the CsSnI_3_ NC solution constant while varying the concentration of the polymer in the same volume. This allowed us to understand the effect of the polymer concentration. The polymers with the heteroatomic functional groups can interact chemically with the perovskite NCs in a synergistic or nonsynergistic manner.^50^ We observed that among the insulating polymers, PS was inert toward the CsSnI_3_ NCs for a test period of 3 days (Figure S4) because no shift and broadening of the excitonic peak was observed in UV–visible absorption spectra. In contrast, the conducting polymers introduced a broadening of the CsSnI_3_ NC absorbance peak and a new peak around ≈400 nm originating from π-conjugation of polymers^51^ in all of the cases, as can be seen in Figure S5.

In general, the variation of the polymer to the CsSnI_3_ NC ratio showed a minimal effect on the absorbance feature of NCs (Figure S6a). A similar observation was derived when the dilution studies of 1:1 (v/v) solution of CsSnI_3_ NCs (≈50 mg/mL) and 2% PS were performed (Figure S6b).

As a result of our polymer screening experiments (Figures S4 and S5), we chose PS, a simple nonheteroatom polymer with a monomer resembling our solvent toluene and successfully used earlier for Pb halide perovskite NCs,^15,49,52,53^ to encapsulate the CsSnI_3_ NC thin film via different methodologies described below.

**Figure 2:**
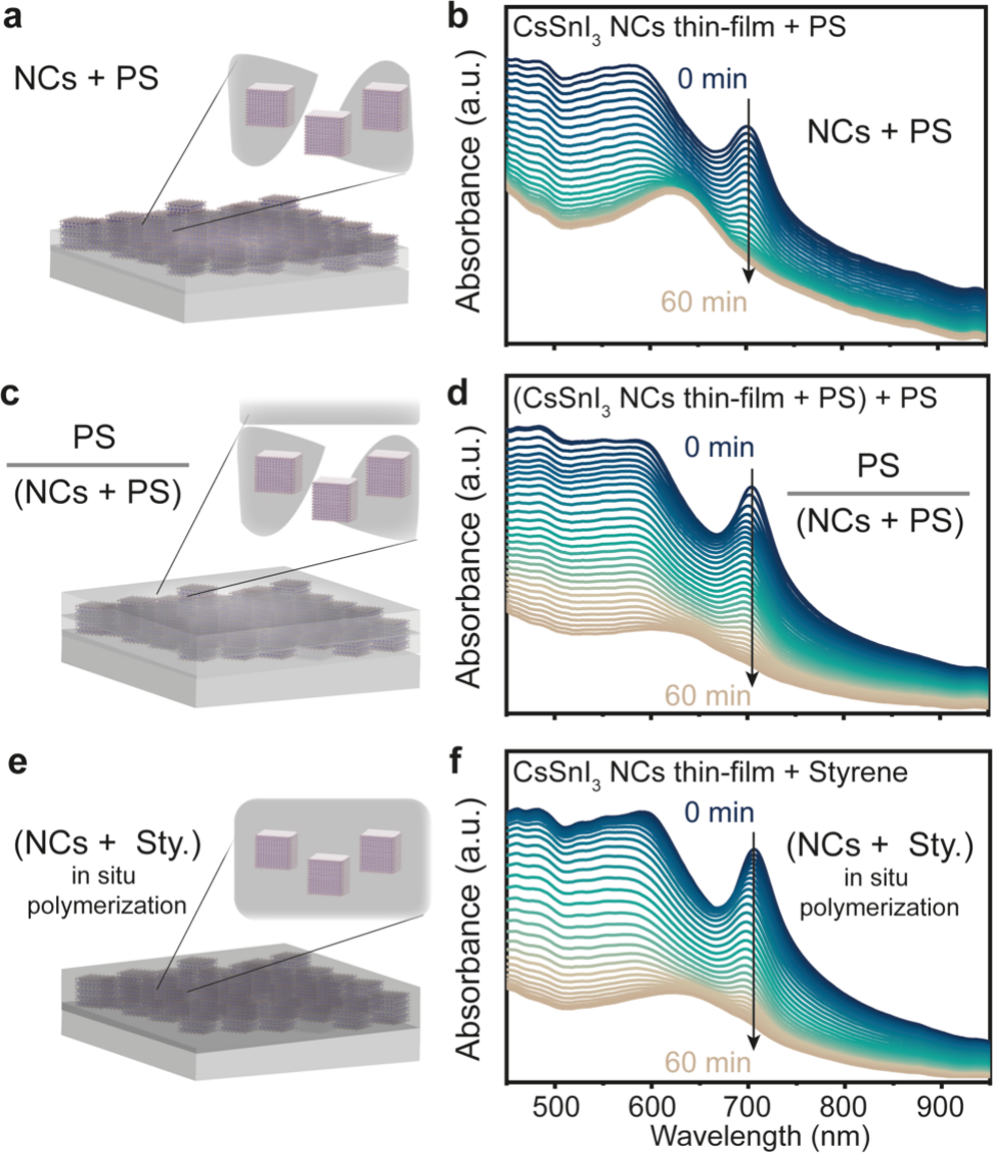
Organic encapsulation of the CsSnI_3_ NC thin film with PS performed using different methodologies. (a) Thin film fabricated via CsSnI_3_ NC/PS = 1:1 solution showing the evolution of (b) UV–visible absorption spectra in the air. (c) Thin film fabricated via a CsSnI_3_ NC/PS = 1:1 solution topped with a layer of PS showing the evolution of (d) UV–visible spectra in the air. (e) In situ polymerization of the CsSnI_3_ NC solution in styrene performed using AIBN at 80 °C for 36–48 h, showing the evolution of (f) UV–visible absorption spectra in the air.

Figure 2 highlights the three different strategies of encapsulation which we probed with the CsSnI_3_ NC film and PS: (i) encapsulation with 1:1 solution of CsSnI_3_ NCs and PS; (ii) encapsulation with 1:1 solution of CsSnI_3_ NCs and PS, which is topped with an extra layer of PS; and (iii) encapsulation with in situ polymerization of styrene. When we fabricated a thin film of CsSnI_3_:PS = 1:1 solution, we observed that the absorption peak at 702 nm decreased relatively slowly as compared to only the NC thin film, which indicated a slower diffusion of oxygen and moisture in the presence of the polymer (Figure 2a,b). However, a slight improvement in the air stability was noticed. This can be due to the presence of CsSnI_3_ NCs exposed toward the air interface. Thus, to provide complete encapsulation to NCs, an extra layer of PS was cast on top of former thin films. This extra PS layer did not help achieve a higher air stability, as shown in Figure 2c,d. After the dissolution of CsSnI_3_ NCs in styrene, we fabricated the thin film and utilized the in situ polymerization process for the encapsulation, as illustrated in Figure 2e (details are provided in the method section above).

Figure 2f shows the evolution of the UV–visible absorption spectra within the air exposure time. We did not observe any enhancement in the air stability of our thin films using the in situ encapsulation procedure. The normalized absorption peak at 702 nm decayed at a similar rate when compared to ex situ encapsulation, as plotted in Figure 2d.

Organic encapsulation methodologies with the PS matrix provided a slight increase in the air stability of the CsSnI_3_ NC thin film compared to the pristine CsSnI_3_ NC thin film (Figure S7). We further compared PS with PMMA and we observed a faster degradation trend (Figure S8) due to the hydrophilic nature of PMMA.^14^

### Inorganic Encapsulation of the CsSnX_3_ NC Thin Film

Inorganic encapsulation of thin films can be performed *via* a variety of deposition techniques such as chemical vapor deposition (CVD), physical vapor deposition (PVD), pulsed laser deposition (PLD), ALD, etc., utilizing a wide spectrum of inorganic materials.^33,54^ For softer materials like metal halide perovskites, ALD of metal oxides, specifically Al_2_O_3_, has proven to be successful in improving the long-term stability and maintaining the integrity of the perovskite structure.^55^ Al_2_O_3_ is highly transparent, electrically insulating, and exhibits a quite low water vapor as well as oxygen transmission rate.^19−21^ Al_2_O_3_ ALD is being increasingly employed in photovoltaics as a protection layer,^56,57^ in field-effect transistors (FETs) as diffusion barriers,^58^ and for the functionalization of polymers^59^ and graphene.^60,61^ Colloidal ALD (c-ALD) of Al_2_O_3_ has also been successfully demonstrated for improving the stability of Pb halide perovskite NCs and their respective superlattices.^62,63^ Therefore, we opted to employ Al_2_O_3_ deposition via the ALD process to improve our CsSnX_3_ NC thin film.

**Figure 3:**
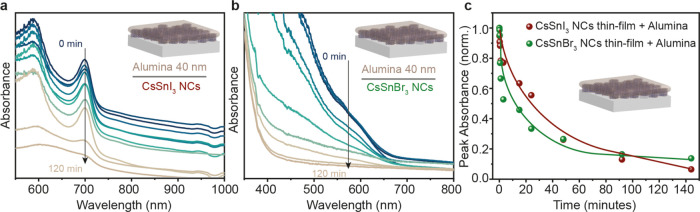
Inorganic encapsulation of the CsSnX_3_ NC thin film with Al_2_O_3_ performed *via* ALD. (a) Evolution of UV–visible absorption spectra of the CsSnI_3_ NC thin film in air with 40 nm Al_2_O_3_ deposition. (b) Evolution of UV–visible absorption spectra of the CsSnBr_3_ NC thin film in air with 40 nm Al_2_O_3_ deposition. (c) Comparison plot for CsSnI_3_ and CsSnBr_3_ NC thin films in air with 40 nm Al_2_O_3_ deposition.

We performed ALD on the pristine CsSnX_3_ NC thin film to grow the Al_2_O_3_ layer with a thickness of 40 nm. Figure 3a,b shows the evolution of UV–visible spectra upon air exposure for the CsSnI_3_ and CsSnBr_3_ NC thin films coated with a 40 nm Al_2_O_3_ layer. The excitonic peaks decreased continually over a time of a couple of hours for both Sn halide perovskite NC systems (Figure 3c). In comparison with organic polymer encapsulation, we observed an enhancement in the air stability of the CsSnX_3_ NC thin film by an hour. Even if these results showed considerable improvement compared with pristine thin films, we concluded that these encapsulation strategies can be used in tandem in a synergistic manner.^11,32,64,65^ This is due to the inorganic component acting as a permeation barrier and the organic component reducing the propagation of the defects and its hydrophobicity, which can help in enhancing the air stability of the CsSnX_3_ NC thin film.^64^

### Organic–Inorganic Hybrid Encapsulation of the CsSnX_3_ NC Thin Film

The hybrid organic–inorganic encapsulation of thin films was already achieved successfully for the long-term stability and integrity of organic optoelectronic/electronic devices.^25,28,29^ Thus, this hybrid encapsulation strategy appeared to be promising for providing a significant blockade to the ambient environment for the CsSnX_3_ NC thin film and improving their air stability.

For the organic–inorganic hybrid encapsulation, we have utilized Al_2_O_3_ ALD over the thin layer of the polymer on the CsSnX_3_ NC thin film. We decided to employ a thin layer of PMMA based on the compatibility of the polymer with 40 nm Al_2_O_3_ in terms of superior adhesion and the presence of stronger chemical interactions due to polar groups, which is illustrated in Figure S8.^66−68^ Note that we performed a 20 nm Al_2_O_3_ thin-film deposition for comparison (Figure S9), but with this thinner inorganic layer, we observed a similar decrease in the absorbance feature as in organic encapsulation methods. Generally, PMMA is employed successfully with Pb halide perovskite bulk thin films and NC thin films in various optoelectronic applications.^36,69^ On the other hand, PS shows a weaker adhesion to Al_2_O_3_ compared to PMMA.

Moreover, with the presence of PMMA over thin films, the Al_2_O_3_ ALD reactions are modified for the initial few cycles. Trimethylaluminum (TMA), being a stronger Lewis acid, forms an adduct with the carbonyl (−C=O) group on PMMA succeeded by methyl transfer to carbon, which forms an acetal unit (Figure S10). With the introduction of water, Al(OH)_2_ units are formed on the surface, which further proceeds *via* a mechanism proposed by George et al.^70^

**Figure 4:**
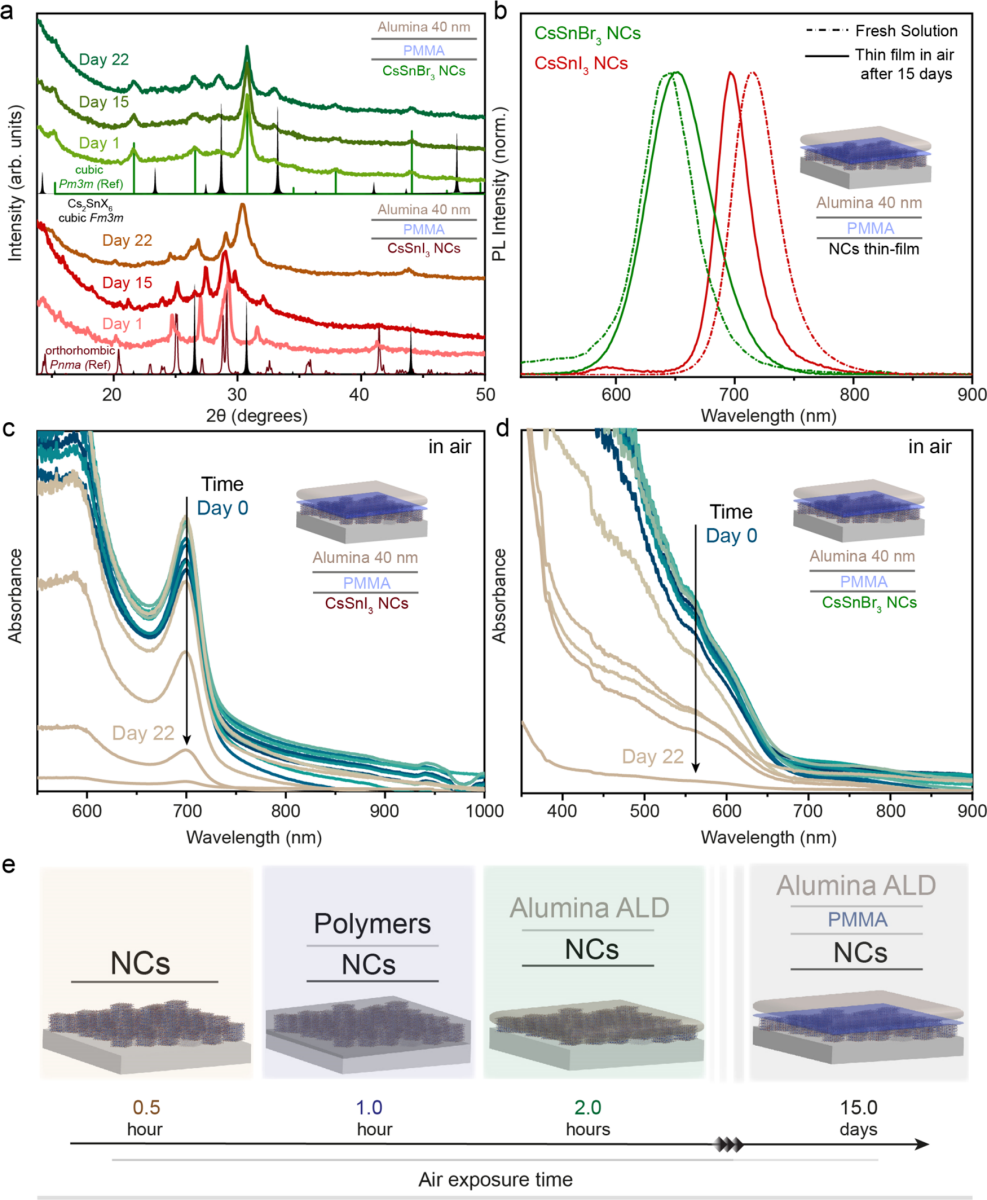
Organic–inorganic hybrid encapsulation of the CsSnX_3_ NC thin film with a thin layer of PMMA and ALD-grown Al_2_O_3_. (a) Evolution of the XRD pattern of the CsSnI_3_ NC/PMMA/Al_2_O_3_ thin film (red-brown) and the CsSnBr_3_ NC/PMMA/Al_2_O_3_ thin film kept in air. (b) Steady-state PL measurements of the CsSnI_3_ NC/PMMA/Al_2_O_3_ thin film (red-brown) and the CsSnBr_3_ NC/PMMA/Al_2_O_3_ thin film (green) after 15 days in air in comparison with the PL of the NC solution (dashed-dotted line). (c) Evolution of the UV–visible absorbance of the CsSnI_3_ NC/PMMA/Al_2_O_3_ thin film in air for 22 days. (d) Evolution of the UV–visible absorbance of the CsSnBr_3_ NC/PMMA/Al_2_O_3_ thin film in air for 22 days. (e) Schematic diagram depicting the improvement in the air stability for various encapsulation strategies employed to the CsSnX_3_ NC thin film.

After the ALD deposition of 40 nm thick Al_2_O_3_ on a PMMA-layered CsSnX_3_ NC thin film, we observed a superior air stability in the obtained thin films. Figure 4a shows the evolution of XRD patterns with the air exposure time for the CsSnI_3_ and CsSnBr_3_ NC thin films. In detail, for CsSnI_3_ NC thin films, we observed that the crystal structure is maintained as orthorhombic *Pnma* over a period of 15 days, which is a record enhancement compared to our own only organic and inorganic encapsulation and with respect to the previous reports.^27,39^ The broadness of the diffraction peaks confirmed the maintenance of the morphology and size of NCs in the thin film. A similar observation was noted for the CsSnBr_3_ NC thin film. Moreover, we observed the PL emission from these thin films even after air exposure of 15 days (Figure 4b). For the CsSnI_3_ NC thin film, the PL emission seems to be blue-shifted from 712 to 700 nm (≈0.03 eV) with respect to the fresh NC dispersion. The appearance of a new PL peak around ≈600 nm shows the degradation toward the low-dimensional 2D Ruddlesden–Popper perovskite structures, which are energetically more favorable to form.^71^ For the CsSnBr_3_ NC thin film, the PL emission after air exposure was red-shifted from 644 to 652 nm (≈0.02 eV) with respect to the fresh dispersion of NCs, which disclosed a slight aggregation, also confirmed by the increase of the fwhm of the XRD pattern (Figure 4b). The NC thin film measured after 15 days started to show the formation of the corresponding oxidized double perovskite composition Cs_2_SnI_6_ (cubic, *Fm*3*m*) (Figure 4a). Figure 4c,d shows the evolution of the absorbance for CsSnI_3_ and CsSnBr_3_ NC thin films, respectively, which aligned well with the degradation timeline observed with the XRD technique. When we performed ALD deposition of 40 nm thick Al_2_O_3_ on the PS-layered CsSnX_3_ NC thin film, we observed a decrease in the absorbance feature within 6 days for CsSnI_3_ and within 4 days for CsSnBr_3_ NC thin films (Figure S11). This also provides evidence of a better adhesion mechanism transpiring with the PMMA-layered CsSnX_3_ NC thin film, leading to enhanced ambient stability.

Following a systematic study, we demonstrated that using the hybrid polymer/Al_2_O_3_ strategy, the structural integrity and optical properties are maintained intact even after 15 days of air exposure for the CsSnX_3_ NC thin film (Figure 4e).

## Conclusions

To enhance the stability of CsSnX_3_ nanocrystal (NC) thin films, we employed three encapsulation methods, namely, organic, inorganic, and hybrid organic–inorganic. While a nominal improvement was observed *via* organic and inorganic encapsulation performed independently, hybrid organic–inorganic encapsulation by depositing a thin layer of PMMA followed by a 40 nm Al_2_O_3_ layer via ALD improved the ambient stability of the CsSnX_3_ NC thin films for up to 15 days, as characterized by temporal X-ray diffraction and optical spectroscopy. This record improvement in air stability marks a significant advancement, facilitating further studies into the structural and physical properties of tin halide perovskite nanocrystals processed in thin films.

## Supporting Information Available

Experimental details; characterization techniques; supporting figures (evolution of PL and UV–visible spectra for bare CsSnI_3_ NCs, encapsulated with different polymers), and schematic figures (PDF)

## Abbreviations

NCs: nanocrystals
ALD: atomic layer deposition
PS: poly(styrene)
PMMA: poly(methyl methacrylate)
PVDF: poly(vinylidene fluoride)
PDMS: polydimethylsiloxane
COC: cyclic olefin copolymer
PLD: pulsed laser deposition
fwhm: full width at half-maximum
PL: photoluminescence
XRD: X-ray diffraction
PFO: poly(dioctylfluorene)
p-TPD: poly-*N*,*N*′-bis(3-methylphenyl)-*N*,*N*′-diphenylbenzidine
PF-12: poly-9,9′-di-*n*-dodecyl-fluorenyl-2,7-diyl
PVK: poly(vinylcarbazole)
AIBN: azobis(isobutyronitrile)
CVD: chemical vapor deposition
PVD: physical vapor deposition
c-ALD: colloidal ALD
