# Supplementary material for: Air-Stable Thin Films of Tin Halide Perovskite Nanocrystals by Polymers and Al2O3 Encapsulation
Source: Chem Mater. 2024 Nov 15;36(22):11227–35. doi: 10.1021/acs.chemmater.4c02261 (PMC11603609; doi:10.1021/acs.chemmater.4c02261)
Supplement: Supplementary file 1 — cm4c02261_si_001.pdf [file cm4c02261_si_001.pdf]

Supporting Information

Air stable Thin Films of Tin-Halide Perovskite Nanocrystals by Polymers and Al_2O_3 Encapsulation

Kushagra Gahlot[a], Lorenzo di Mario[a], Rixt Bosma[a], Maria A. Loi[a] and Loredana Protesescu[a,]*

[a]Zernike Institute for Advanced Materials, University of Groningen, Nijenborgh 4, 9747 AG, The Netherland. Email – l.protesescu@rug.nl

Table of contents

	Description	Page
	Supporting figures	3-10
**Figure S1**	Scanning Transmission Electron Microscopy (STEM) images for $CsSnI_3$ and $CsSnBr_3$ NCs at different magnifications.	3
**Figure S2**	Evolution of PL emission spectra on the air exposure for $CsSnI_3$ NCs solution and thin-film.	4
**Figure S3**	General schematic for the procedure of $CsSnI_3$ NCs/polymer solution for the thin-film fabrication.	4
**Figure S4**	Evolution of UV-visible absorbance spectra for $CsSnI_3$ NCs solution with different insulating polymers.	5
**Figure S5**	Evolution of UV-visible absorbance spectra for $CsSnI_3$ NCs solution with different conducting polymers.	6
**Figure S6**	Evolution of UV-visible absorbance spectra for $CsSnI_3$ NCs solution with different concentrations of PS and sequential dilution.	7
**Figure S7**	Normalized peak absorbance Vs. time (minutes) plot for organic encapsulation cases in comparison with only NCs	7
**Figure S8**	Evolution of UV-visible absorbance spectra of PMMA encapsulated $CsSnI_3$ NCs thin-film	8
**Figure S9**	Evolution of UV-visible absorbance spectra for $CsSnI_3$ NCs thin film encapsulated with 20 nm thick alumina.deposition.	8
**Figure S10**	Illustration of chemical interactions at the PMMA/alumina interface with proposed reaction mechanism	9
**Figure S11**	Evolution of UV-visible absorbance of $CsSnI_3$ NCs/PS/Al_2O_3 thin-film (a) and $CsSnBr_3$ NCs/PS/Al_2O_3 thin-film kept in air.	10
	References	10

Supporting figures

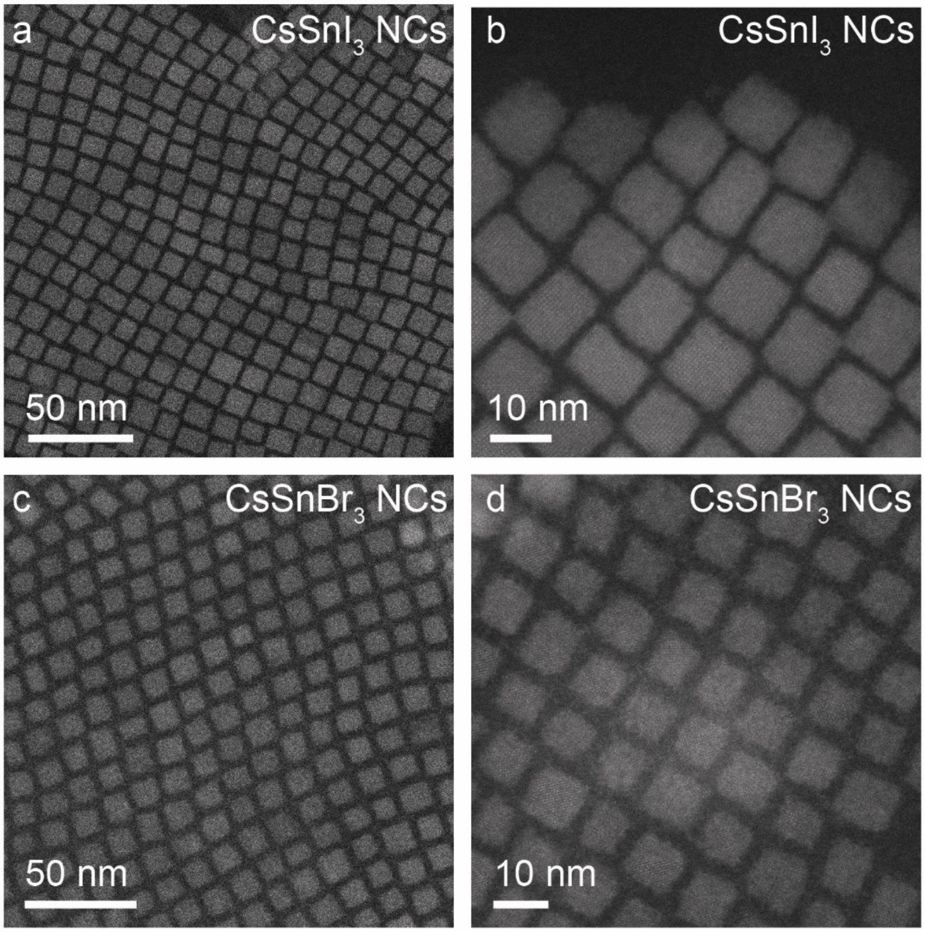

Figure S1. Scanning Transmission Electron Microscopy (STEM) images for $CsSnI_3$ NCs (a, b) and $CsSnBr_3$ NCs (c, d) at different magnifications.

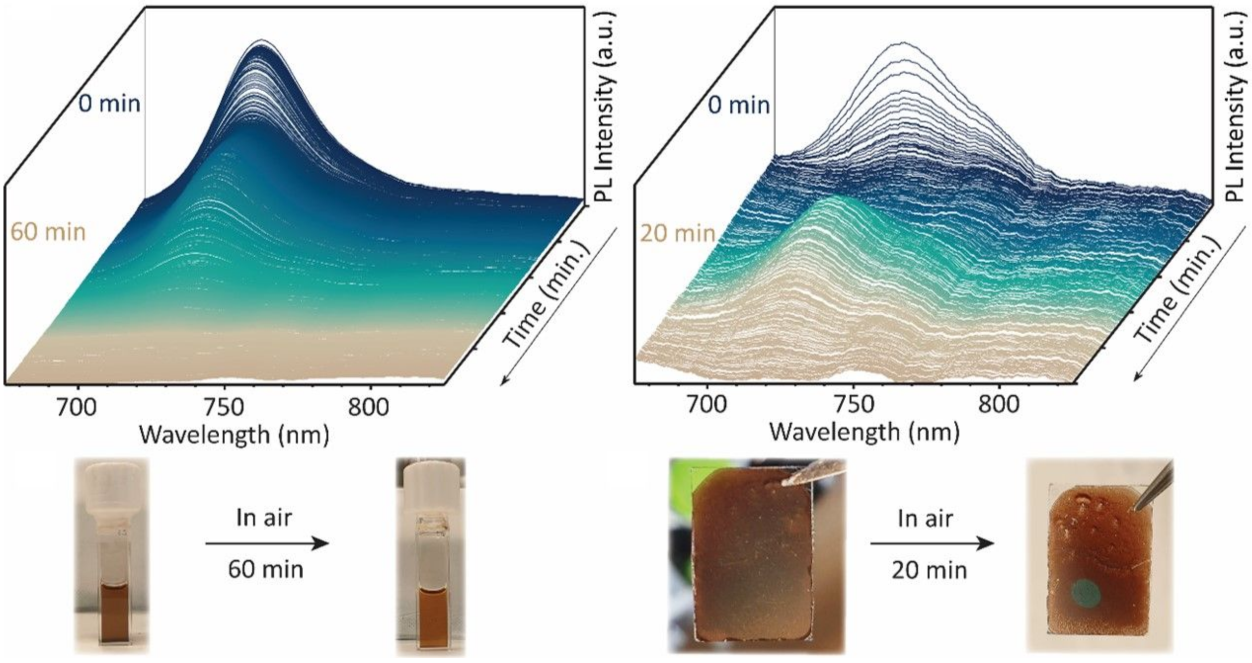

Figure S2. Evolution of PL emission spectra on the air exposure for $CsSnI_3$ NCs solution and thin-film.

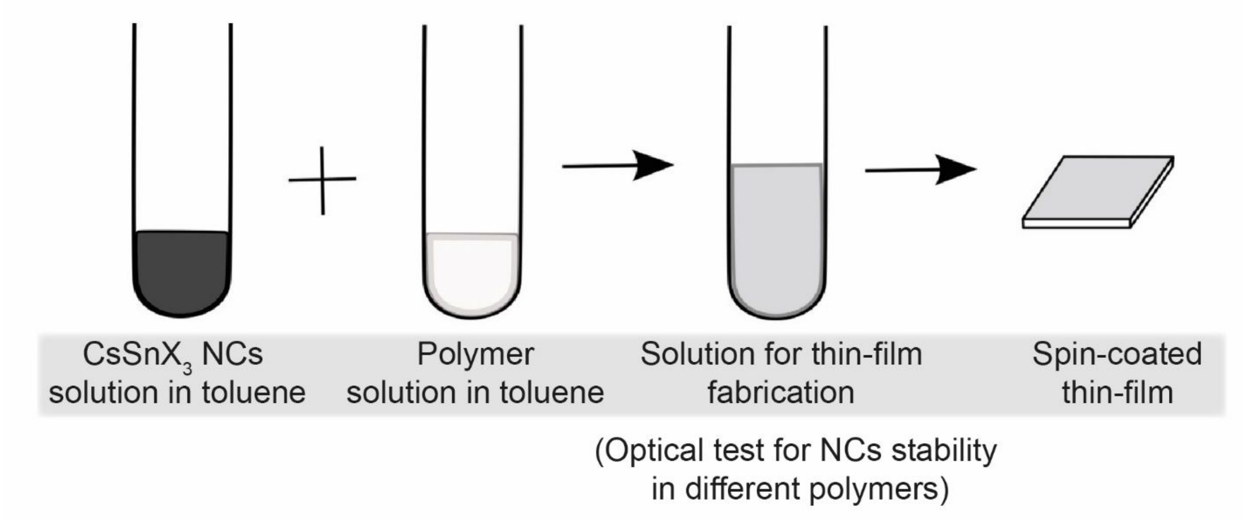

Figure S3. General schematic for the procedure of $CsSnI_3$ NCs/polymer solution for the thin-film fabrication.

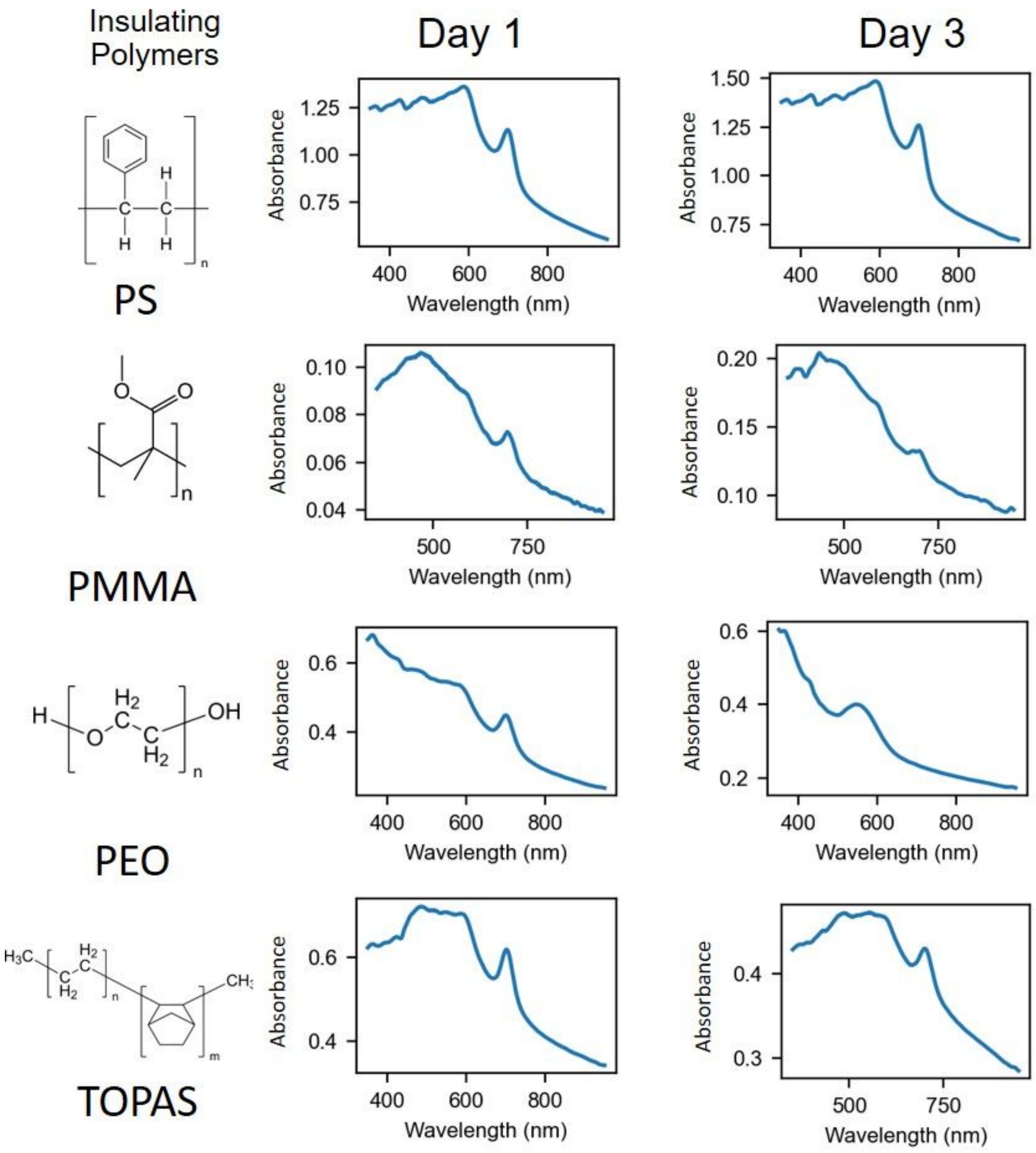

Figure S4. Evolution of UV-visible absorbance spectra for $CsSnI_3$ NCs solution with different insulating polymers with their chemical structure kept in the glove box for three days.

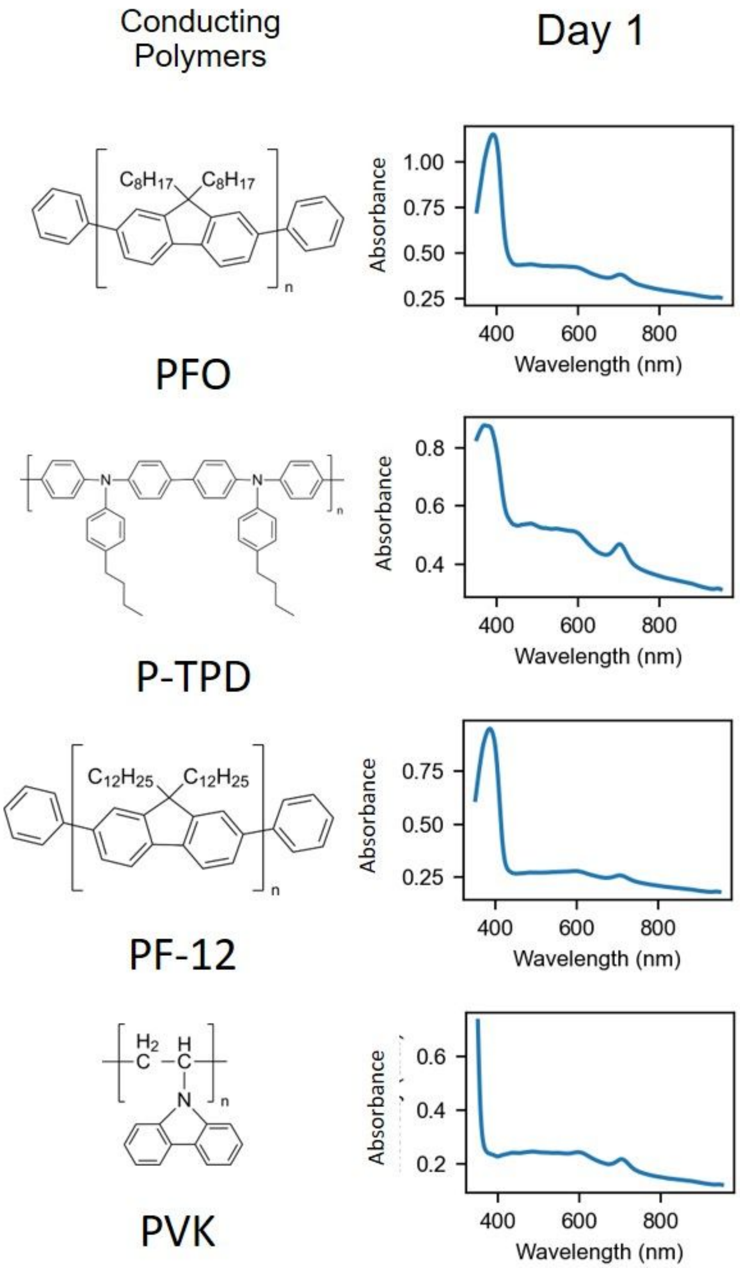

Figure S5. Evolution of UV-visible absorbance spectra for $CsSnI_3$ NCs solution with different conducting polymers with their chemical structure kept in the glove box for three days.

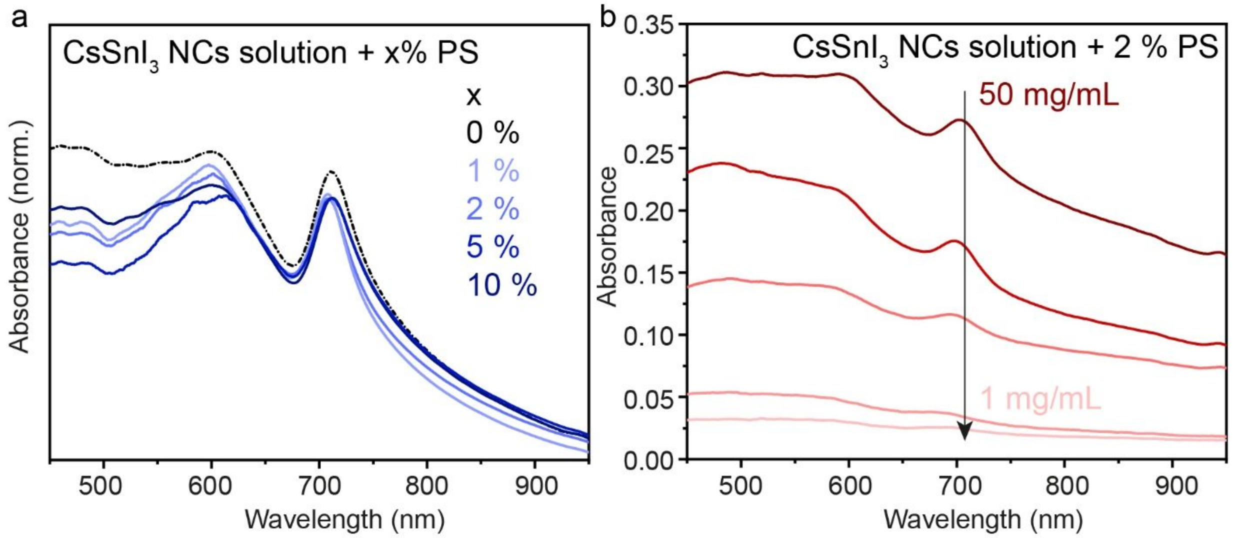

Figure S6. (a) Evolution of UV-visible absorbance spectra for $CsSnI_3$ NCs solution with different concentrations of PS. (b) Evolution of UV-visible absorbance spectra for $CsSnI_3$ NCs solution in sequential dilution.

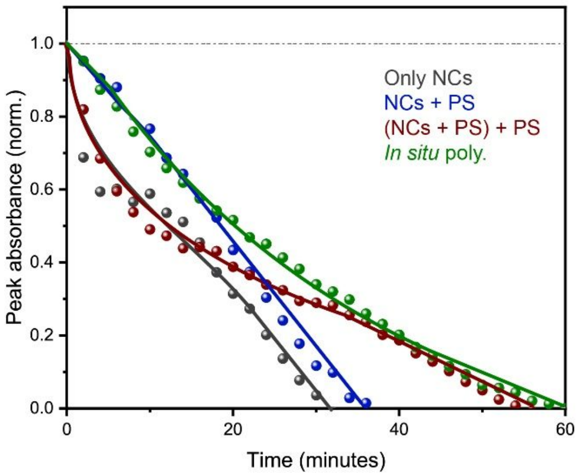

Figure S7. Normalized peak absorbance (λ_{abs} = 702 nm) Vs. time (minutes) plots for organic encapsulation cases in comparison with only NCs (dark grey).

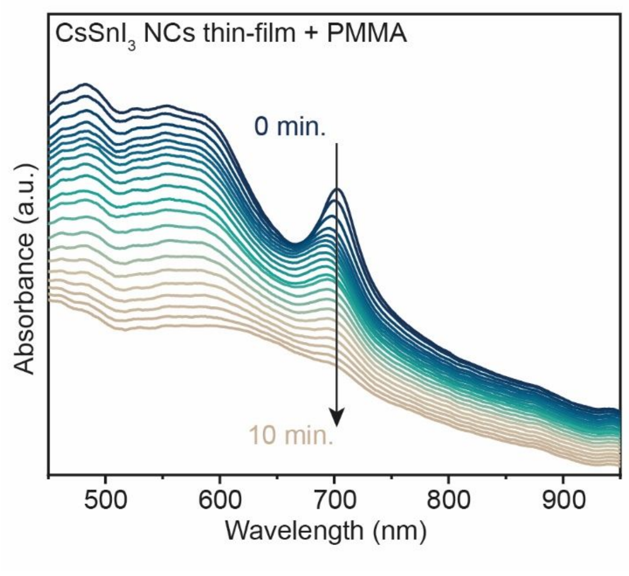

Figure S8. (a) Organic encapsulation of $CsSnI_3$ NCs thin-film with PMMA ($CsSnI_3$ NCs: PMMA = 1: 1 solution, similar to PS) showing the evolution of UV-visible absorbance spectra in air for 10 minutes.

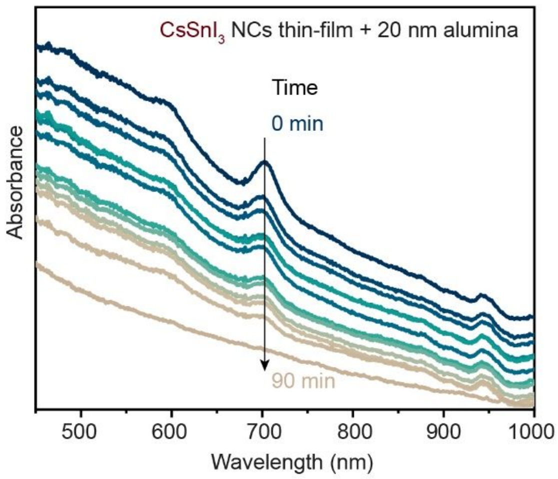

Figure S9. Evolution of UV-visible absorbance spectra for $CsSnI_3$ NCs thin film encapsulated with 20 nm thick alumina.deposition.

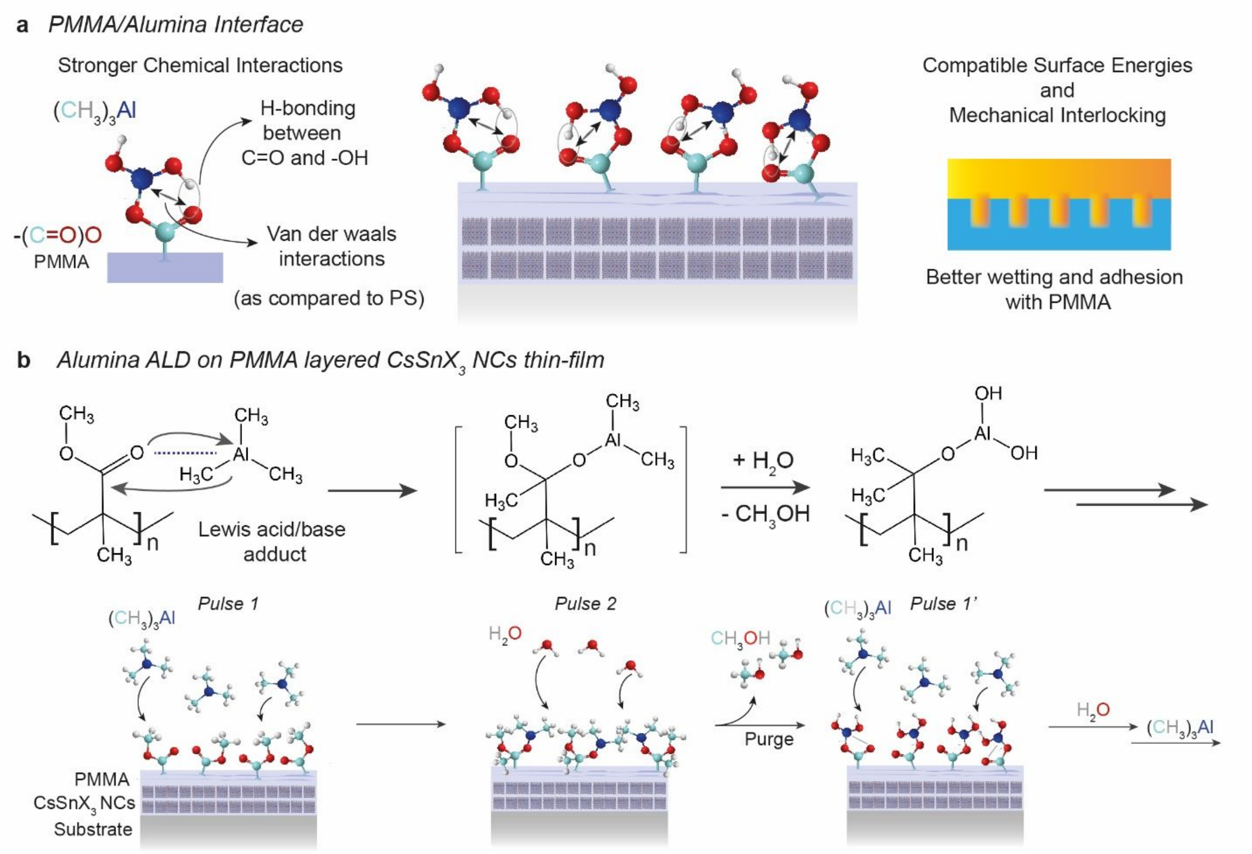

Figure S10. (a) Illustration of favourable interactions occurring at the PMMA/alumina interface contrary to PS. (b) Proposed reaction scheme of alumina ALD over the PMMA encapsulated $CsSnX_3$ NC thin-films leading to effective encapsulation for air-stability.[1]

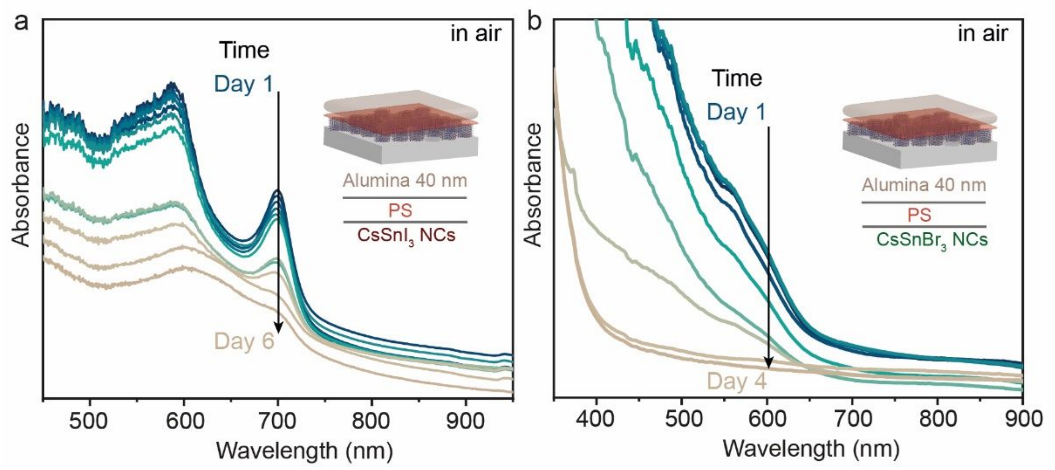

Figure S11. Evolution of UV-visible absorbance of $CsSnI_3$ NCs/PS/Al_2O_3 thin-film (a) and $CsSnBr_3$ NCs/PS/Al_2O_3 thin-film kept in air.

References

(1) George, S. M. Atomic Layer Deposition: An Overview. *Chem. Rev.* **2010**, *110* (1), 111-131.
